# Supplementary material for: Divergent evolution of Corynebacterium diphtheriae in India: An update from National Diphtheria Surveillance network
Source: PLoS One. 2021 Dec 15;16(12):e0261435. doi: 10.1371/journal.pone.0261435 (PMC8673651; doi:10.1371/journal.pone.0261435)
Supplement: S1 Table — (DOCX) [file pone.0261435.s001.docx]

**S1 Table:** Laboratory confirmation of *C. diphtheriae* cases at NRL for diphtheria, CMC, Vellore

| **Centres** | **Specimen**  **type** | **Specimen Received** | **Specimen numbers** | **Culture positives from samples** | ***C. diphtheriae* (*rpo*B)** | ***tox*A** | ***Culture negative PCR positive*** | ***Toxin negatives*** |
| --- | --- | --- | --- | --- | --- | --- | --- | --- |
| CMC, Vellore, TN | Throat swab | 2015 | 25 | 2 | 2 | 1 | 0 | 1 |
| CMC, Vellore, TN | Throat swab | 2016 | 42 | 7 | 7 | 6 | 0 | 1 |
| CMC, Vellore, TN | Throat swab | 2017 | 44 | 5 | 6 | 5 | 1 | 1 |
| CMC, Vellore, TN | Throat swab | 2018 | 79 | 14 | 27 | 24 | 13 | 3 |
| CMC, Vellore, TN | ear, nasal swab, pus, tissue | 2016 | 6 | 1 | 4 | 0 | 3 | 4 |
| CMC, Vellore, TN | ear, nasal swab, pus, tissue | 2017 | 7 | 1 | 2 | 1 | 1 | 1 |
| CMC, Vellore, TN | ear, nasal swab, pus, tissue | 2018 | 6 | 3 | 4 | 2 | 1 | 2 |
| Coimbatore Medical College, TN | Isolate | 2017 | 7 | 7 | 7 | 7 | 0 | 0 |
| Coimbatore Medical College, TN | Isolate | 2018 | 8 | 8 | 8 | 8 | 0 | 0 |
| State Public Health, Trivandrum, Kerala | Isolate | 2016 | 42 | 24 | 24 | 22 | 0 | 2 |
| State Public Health, Trivandrum, Kerala | Isolate | 2017 | 47 | 47 | 47 | 37 | 0 | 10 |
| State Public Health, Trivandrum, Kerala | Isolate | 2019 | 4 | 4 | 4 | 4 | 0 | 0 |
| St. John’s Hospital, Karnataka | Isolate | 2017 | 2 | 2 | 2 | 2 | 0 | 0 |
| S. Nijalingappa Medical College, Karnataka | Isolate | 2017 | 1 | 1 | 1 | 1 | 0 | 0 |
| S. Nijalingappa Medical College, Karnataka | Isolate | 2018 | 9 | 9 | 9 | 9 | 0 | 0 |
| KMC, Manipal,Karnataka | Isolate | 2018 | 1 | 1 | 1 | 1 | 0 | 0 |
| Calicut | Isolate | 2016 | 1 | 1 | 1 | 1 | 0 | 0 |
| Global Hospitals, Hyderabad | Isolate | 2017 | 1 | 1 | 1 | 1 | 0 | 0 |
| Madurai Medical College | Isolate | 2018 | 3 | 3 | 3 | 3 | 0 | 0 |
| KGMC, Lucknow | Isolate | 2018 | 34 | 30 | 30 | 30 | 0 | 0 |
| Bharathi Vidhyapeeth, Sangli | Isolate | 2018 | 7 | 7 | 7 | 7 | 0 | 0 |
| KKCTH, Chennai | Throat swab | 2018 | 27 | 3 | 10 | 5 | 7 | 5 |
| KKCTH, Chennai | Isolate | 2018 | 1 | 1 | 1 | 0 | 0 | 1 |
| Kauvery Hopsital, Trichy | Isolate | 2018 | 1 | 1 | 1 | 0 | 0 | 1 |
| Tirunelveli Med. College,Tirunelveli | Isolate | 2018 | 1 | 1 | 1 | 1 | 0 | 0 |
| Govt. Mohan Kumaramangalam Med. College, Salem | Isolate | 2018 | 6 | 6 | 6 | 6 | 0 | 0 |
| LTM Medical College, Mumbai | Isolate | 2018 | 1 | 1 | 1 | 1 | 0 | 0 |
| LTM Medical College, Mumbai | Throat swab | 2018 | 1 | 1 | 1 | 1 | 0 | 0 |
| Dr.Shankaroa Chavan Gov. Med. College, Vishnupuri, Nanded, Maharashtra | Isolate | 2018 | 9 | 7 | 7 | 7 | 0 | 0 |
| RIMS, Raichur | Isolate | 2018 | 11 | 11 | 11 | 11 | 0 | 0 |
| Southern Railway Hospital, Chennai | Isolate | 2018 | 1 | 1 | 1 | 0 | 0 | 1 |
| PGIMER, Chandigarh | Isolate | 2019 | 1 | 1 | 1 | 1 | 0 | 0 |
| PGIMER, Chandigarh | Isolate | 2020 | 5 | 5 | 5 | 5 | 0 | 0 |
| **Total** |  |  | **441** | **217** | **243** | **210** | **26** | **33** |
