# Supplementary material for: Divergent evolution of Corynebacterium diphtheriae in India: An update from National Diphtheria Surveillance network
Source: PLoS One. 2021 Dec 15;16(12):e0261435. doi: 10.1371/journal.pone.0261435 (PMC8673651; doi:10.1371/journal.pone.0261435)
Supplement: S2 Table — (DOCX) [file pone.0261435.s002.docx]

**S2 Table:** Distribution of sequence types among different states in India

|  | **Tamil Nadu**  **(n = 35)** | **Karnataka**  **(n = 22)** | **Andhra Pradesh**  **(n = 7)** | **Kerala**  **(n = 77)** | **Uttar Pradesh**  **(n = 46)** | **Madhya Pradesh (n = 4)** | **Maharashtra (n = 3)** | **Chandigarh (n = 14)** | **Delhi**  **(n = 6)** | **Bangladesh**  **(n = 1)** | **Total** |
| --- | --- | --- | --- | --- | --- | --- | --- | --- | --- | --- | --- |
| ST50 | 6 | 12 | 2 | 2 | 1 |  |  |  |  |  | 23 |
| ST295 |  |  | 1 | 1 |  |  |  |  |  |  | 2 |
| ST301 |  |  |  |  | 14 |  |  | 2 |  |  | 16 |
| ST308 |  |  |  | 1 |  |  |  |  |  |  | 1 |
| ST377 | 3 |  | 2 | 11 |  |  |  | 1 | 3 |  | 20 |
| **ST405** | 10 | 7 | 2 | 8 | 1 |  | 1 | 2 |  |  | 31 |
| **ST408** | 2 |  |  | 3 |  |  |  |  |  |  | 5 |
| **ST409** |  |  |  |  |  |  |  |  |  | 1 | 1 |
| **ST422** |  |  |  | 1 |  |  |  |  |  |  | 1 |
| **ST443** |  |  |  | 1 |  |  |  |  |  |  | 1 |
| **ST446** |  | 1 |  |  |  |  |  |  |  |  | 1 |
| **ST466** | 1 | 1 |  | 23 | 22 | 3 | 2 | 10 | 2 |  | 64 |
| **ST468** |  |  |  | 1 |  |  |  |  |  |  | 1 |
| **ST469** | 3 |  |  | 13 |  |  |  |  |  |  | 16 |
| **ST470** |  |  |  | 1 |  |  |  |  |  |  | 1 |
| **ST540** |  |  |  | 1 |  |  |  |  |  |  | 1 |
| **ST541** |  |  |  | 1 |  |  |  |  |  |  | 1 |
| **ST542** | 1 |  |  | 3 |  |  |  |  |  |  | 4 |
| **ST548** |  |  |  |  | 1 | 1 |  |  |  |  | 2 |
| **ST566** |  |  |  | 1 |  |  |  |  |  |  | 1 |
| **ST567** |  |  |  | 1 |  |  |  |  |  |  | 1 |
| **ST568** |  |  |  | 1 |  |  |  |  |  |  | 1 |
| **ST569** |  |  |  | 3 |  |  |  |  |  |  | 3 |
| **ST570** | 4 |  |  |  |  |  |  |  |  |  | 4 |
| **ST573** |  |  |  |  | 1 |  |  |  |  |  | 1 |
| **ST574** |  |  |  |  | 1 |  |  |  |  |  | 1 |
| **ST575** | 2 |  |  |  |  |  |  |  |  |  | 2 |
| **ST576** | 1 | 1 |  |  | 1 |  |  |  |  |  | 3 |
| **ST587** |  |  |  |  | 1 |  |  |  |  |  | 1 |
| **ST588** |  |  |  |  | 1 |  |  |  |  |  | 1 |
| **ST590** |  |  |  |  | 1 |  |  |  |  |  | 1 |
| **ST591** | 1 |  |  |  | 1 |  |  |  |  |  | 2 |
| **ST592** |  |  |  |  |  |  |  |  | 1 |  | 1 |
| **ST599** | 1 |  |  |  |  |  |  |  |  |  | 1 |

**STs in bold are new sequence types reported from this surveillance data.**
