# Supplementary material for: Divergent evolution of Corynebacterium diphtheriae in India: An update from National Diphtheria Surveillance network
Source: PLoS One. 2021 Dec 15;16(12):e0261435. doi: 10.1371/journal.pone.0261435 (PMC8673651; doi:10.1371/journal.pone.0261435)
Supplement: Attachment — Submitted filename: Response to reviewers.docx [file pone.0261435.s004.docx]

**Response to reviewer comments**

I have a few comments to the authors:

1. the data statement says all the data is available, but I cannot find links to the MLST profiles or teh data used in the iTOL tree....

Data for all individual isolates included in the iTOL tree are now given in supplementary table 3. Line 161-162.

2. How related are the strains described in this manuscript to those in Will et al 2021 (Nat Comms) there is overlap in the authorship and it is important that the strains with whole genomes can be cross referenced – this must be stated.

The current study does not represent whole genome data and it primarily discusses the epidemiological stand of *C. diphtheriae* in India. However, citation to will et al is included to refer detailed information on few isolates from this study included in study by Will et al.

3. It would be good to have more international context too….such as relationships to other Asian strains and the reference to global strains in the text (around the text lines 244-253) – is it possible to add some date context to these…and potentially speculate on the emergence of these in India? Where they introduced to india? Or was india the source of these clones globally….

More than 70% of the isolates observed in India are found to be novel STs and are not reported elsewhere. MLST data is not sufficient to speculate origin of existing STs. It needs complete genome information from global data set to compare with the Indian isolates, which itself is a separate study. However, to provide a global representation, previous information on existing STs observed in India is included in the discussion as suggested by the reviewer.

Line 63 – ‘India reported the highest number of diphtheria cases 64 globally’
…this sentence should be contextualised either with a date ‘India reported the highest number of diphtheria cases 64 globally between 2015 and 2020’ [if that is correct?]
or made open ended ‘India reports the highest number of diphtheria cases 64 globally’

The statement has been modified to include details on the number of cases reported as suggested. “India reported the highest number of diphtheria cases globally as of October 2020”.

line 97/98 the unknown reason for why vaccinated individuals become affected…such as waning immunity or strain variation…

The sentence has been modified as “However, fully vaccinated individuals also get affected for unknown reasons including waning immunity, personal health, nutrition status, and infection due to non-toxigenic or variant strains”. Line 97-99.

line 318 – please provide PubMLST link and this also probably needs the link to Bolt et al for the primers references…

The link for PubMLST is now updated and included reference for Bolt et al. 2010 as suggested (Line 283).
