# Supplementary material for: Divergent evolution of Corynebacterium diphtheriae in India: An update from National Diphtheria Surveillance network
Source: PLoS One. 2021 Dec 15;16(12):e0261435. doi: 10.1371/journal.pone.0261435 (PMC8673651; doi:10.1371/journal.pone.0261435)
Supplement: Attachment — Submitted filename: Response to reviewers.docx [file pone.0261435.s005.docx]

**Response to reviewer comments**

Reviewer #1: the authors have addressed most of the comments - however there are still a couple of things that are still not clear

“India reported the highest number of diphtheria cases globally as of October 2020” - this needs a date range and citation or further explanation...it currently reads like the most diphtheria cases globaly ever have occured in india up to october 2020 - it is important to state this as as it can be used to raise awareness and to obtain funding for the whole community.

The statement has been modified as “According to WHO, India reported the highest number of diphtheria cases with more than half of the overall global diphtheria cases between 1980 and 2019”. Citation (Source: WHO vaccine-preventable diseases: monitoring system 2020 global summary) for the same is included in the introduction section (line 93-95).

Also the data files used to create the iTOL tree should be available as it will be difficult to look at in close detail once printed.

Data labelled in the figure 2 using iTOL is provided as the supplementary table 3.

The overlap of strains in the Will et al., paper should also be explicitly stated as it will enable those genomes to be reanalysed

We thank the reviewer for pointing this. We have included the strain IDs in the supplementary table file S3. These isolate IDs can be used by the readers to refer corresponding genomes mentioned in Will et al., paper.
